# Outcomes of lung cancer surgery in patients with COVID-19 history: a single center cohort study

**DOI:** 10.1007/s11748-022-01871-x

**Published:** 2022-09-14

**Authors:** Piotr Gabryel, Dominika Zielińska, Piotr Skrzypczak, Magdalena Sielewicz, Alessio Campisi, Mariusz Kasprzyk, Cezary Piwkowski

**Affiliations:** 1 Department of Thoracic Surgery, Poznan University of Medical Sciences, Szamarzewskiego 62 Street, 60-569 Poznań, Poland; 2 Department of Thoracic Surgery, University and Hospital Trust–Ospedale Borgo Trento, Piazzale Aristide Stefani 1, Verona, Italy

**Keywords:** Lung cancer, Surgery, Treatment, COVID-19, SARS-CoV-2

## Abstract

**Objectives:**

Coronavirus disease 2019 (COVID-19) can irreversibly damage the lungs and could possibly increase the risk of surgical treatment of lung cancer. The study aimed to assess the relationship between preoperative COVID-19 and complications and early outcomes of lung cancer surgery.

**Methods:**

This single-center cohort study included 444 patients who underwent lobectomy or segmentectomy for primary lung cancer from January 1, 2019 to December 31, 2021. The exclusion criteria were pneumonectomy, extended resection, and wedge resection. The groups of patients with (*n* = 72) and without (*n* = 372) the history of COVID-19 prior to surgery were compared. The groups were similar in terms of distribution of baseline, surgical and histopathological characteristics. The primary endpoint was the incidence of postoperative complications. Secondary endpoints were outcomes at discharge and at 90 days.

**Results:**

The incidence of postoperative complications did not differ between the groups of patients with and without COVID-19 history (30.6% vs 29.3%, *p* = 0.831). Outcomes at discharge and at 90 days after surgery did not differ between the groups. Among the patients with and without prior COVID-19, 97.2 and 99.5% were alive at discharge (*p* = 0.125), and 97.2% and 98.1% ninety days after surgery (*p* = 0.644), respectively. Patients with COVID-19 history more often required re-drainage (6.9% v 2.2%, *p* = 0.044) and reoperation (5.6 v 1.3%, *p* = 0.042).

**Conclusions:**

COVID-19 history is not related to the general incidence of complications, outcomes at discharge from the hospital, and at 90-days after surgery.

## Introduction

According to the World Health Organization data, lung cancer is currently one of the most common cancers and the leading cause of cancer death worldwide [1]. Complete anatomical resection, preferably thoracoscopic lobectomy or segmentectomy, performed at an early stage of the disease, allows for the best long-term treatment results [2]. Despite the progress made in recent years in qualification for surgery, video-assisted thoracic surgery (VATS), and enhanced recovery after surgery (ERAS) programs, approximately one-third of patients experience postoperative complications [3, 4]. So far, many factors related to the incidence of postoperative complications have been identified, such as age, sex, comorbidities, pulmonary function, surgical approach and extent of lung resection [5]. Among these factors, diseases of the cardiovascular and respiratory systems, are the most important [6, 7].

The first cases of SARS-CoV-2 infections were found in China in 2019, and the disease quickly turned into a pandemic. To date, more than 500 million cases have been diagnosed worldwide, including many patients treated for lung cancer [8, 9].

Although SARS-CoV-2 infects most organs in the body, the respiratory system is generally the most affected. Studies have shown that SARS-CoV-2 can lead to highly heterogeneous lung damage [10], which may result in a wide range of clinical symptoms [11] and radiographic abnormalities [12]. In many patients, lung damage may be permanent [13].

We suspect that, as in the case of other serious respiratory diseases, COVID-19 and the lung damage caused by the disease may affect the early outcomes of lung cancer surgery, including postoperative complications. To our knowledge, only case reports and short case-series were published on the subject up to date [14, 15].

The current study aimed to determine whether the COVID-19 history was related to the incidence of complications, and in-hospital and 90-day mortality of non-small cell lung cancer surgery.

## Subjects and methods

The Bioethics Committee of the Poznan University of Medical Sciences waived the need for ethics approval and the need to obtain consent for the collection, analysis, and publication of the retrospectively obtained and anonymized data for this non-interventional study.

This retrospective cohort study included patients who underwent anatomical lung resection for non-small cell lung cancer (NSCLC) in one institution from January 1, 2019 to December 31, 2021. The exclusion criteria were pneumonectomy, wedge resection, extended resection (including chest wall, diaphragm, superior vena cava, arterial and bronchial sleeve), small-cell lung cancer, metastases, and nonneoplastic histology. The study group (PREOP-COV group) consisted of patients with a history of SARS-CoV-2 infection before the surgery. In all these patients, the SARS-CoV-2 infection had been confirmed by the nasopharyngeal swab polymerase chain reaction test. The control group (NON-COV group) included patients who did not have SARS-CoV-2 infection. To avoid unintentional inclusion in the control group of patients who had asymptomatic, undiagnosed SARS-CoV-2 infection before lung surgery, the control group included only patients operated on before the onset of the COVID-19 pandemic in Poland, i.e., from January 1, 2019 to February 29, 2020.

During the preoperative workup, all patients underwent chest computed tomography (CT) scan, abdominal ultrasound, electrocardiography, and pulmonary function test with the calculation of predicted postoperative FEV1% (ppFEV1%), fiberoptic bronchoscopy and positron emission tomography/computed tomography (PET–CT). The perioperative risk was assessed according to the Thoracic Revised Cardiac Risk Index (ThRCRI) and EuroLung1 Risk Score. Charlson Comorbidity Index (CCI) was calculated. The severity of COVID-19 was classified according to National Institute of Health guidelines [16].

Surgery was performed under general anesthesia with double-lumen intubation and single-lung ventilation, using VATS approach or anterolateral thoracotomy. Pulmonary vessels, bronchi, and interlobar fissures were divided with staplers. In some cases, ligatures or vascular clips were used. Systematic mediastinal lymph node dissection or sampling was performed. One 24F chest tube was left in the pleural cavity and connected to an electronic drainage system. The chest drain was removed after resolution of the air leak and when the fluid volume was < 250 mL for 24 h. Follow-up was carried out by the thoracic surgery and oncology outpatient clinics.

### Statistical analysis

Data manipulation and all calculations were performed in IBM^®^ SPSS Statistics^®^ Statistics version 27th (PS Imago Pro 8). Categorical data were analyzed with the Chi-square test or the Fisher–Freeman–Halton test. The unpaired t-test was used to analyze the data with normal distribution and homogeneous variances. The normality of the distribution was tested with the Shapiro–Wilk test, and the equality of variances was checked with Levene’s test. The data that did not follow a Gaussian distribution were analyzed with the Mann–Whitney *U* test. All results were considered significant at a *p* value below 0.05.

## Results

The study included 444 patients after lobectomy or segmentectomy for NSCLC who met all study criteria. The study group (patients who had COVID-19 before surgery, PREOP-COV group) and the control group (patients who did not have SARS-CoV-2 infection, NON-COV group) comprised 72 and 372 patients, respectively. The groups were similar in terms of distribution of baseline, surgical, and histopathological characteristics, including age, sex, BMI, ppFEV1%, comorbidities, ThRCRI, CCI and EuroLung1 scores, surgical approach and type of surgery, histological type, and pathological stage of lung cancer (Tables 1 and 2).

**Table 1:** Comparison of baseline characteristics between patients with (PREOP-COV) and without (NON -COV) history preoperative SARS-CoV-2 infection

Variables^a^	PREOP-COV *n* = 72	NON –COV *n* = 372	*p* value
Age, years	68 (IQR, 63–72)	67 (IQR, 61–72)	0.201
Sex			0.770
Male	43 (59.7)	229 (61.6)	
Female	29 (40.3)	143 (38.9)	
BMI, kg/m^2^	26.7 (IQR, 24.3–30.1)	26.2 (IQR, 23.1–29.4)	0.267
ppFEV_1_%	61.0 (IQR, 53.7–75.8)	64.4 (IQR, 51.3–72.2)	0.315
Comorbidities	61 (84.7)	287 (77.2)	0.153
Chronic obstructive pulmonary disease	17 (23.6)	114 (30.6)	0.231
Coronary arterial disease	15 (13.7)	51 (20.8)	0.120
Cerebrovascular disease	1 (1.4)	13 (3.5)	0.710
Peripheral arterial disease	2 (2.8)	34 (9.1)	0.070
Diabetes mellitus	15 (20.8)	81 (21.8)	0.859
Chronic kidney disease	2 (2.8)	12 (3.2)	0.833
Neoplasms	11 (15.3)	59 (15.9)	0.901
Thoracic revised cardiac risk index			0.668
Class A	54 (75)	311 (83.6)	
Class B	17 (23.6)	58 (15.6)	
Class C	1 (1.4)	3 (0.8)	
Charlson comorbidity index	3 (IQR, 2–4)	3 (IQR, 2–4)	0.899
EuroLung1 score	6 (IQR, 6–9)	6 (IQR, 6–9)	0.987

*BMI* body mass index, *ppFEV*_*1*_*%* predicted postoperative percentage of calculated forced expiratory volume in 1 s

^a^Data are expressed as number (percent), mean ± standard deviation or median (interquartile range)

**Table 2:** Comparison of surgical and histopathological characteristics between patients with (PREOP-COV) and without (NON -COV) history preoperative SARS-CoV-2 infection

Variables^a^	PREOP-COV *n* = 72	NON-COV *n* = 372	*p* value
Surgical approach			0.286
VATS	55 (76.4)	261 (70.2)	
Thoracotomy	17 (23.6)	111 (29.8)	
Pleural adhesions	19 (26.4)	85 (22.9)	0.516
Conversion of VATS to thoracotomy	8 (14.5)	31 (11.9)	0.585
Type of surgery			0.269
Lobectomy	62 (86.1)	324 (87.1)	
Bilobectomy	6 (8.3)	25 (6.7)	
Segmentectomy	4 (5.6)	23 (6.2)	
Estimated blood loss, mL	150 (IQR, 100–250)	150 (IQR, 50–200)	0.007*
Histology			0.762
Adenocarcinoma	39 (54.1)	178 (47.8)	
Squamous cell carcinoma	22 (30.6)	131 (35.2)	
Other cancer	11 (15.3)	63 (17.0)	
Pathological stage			0.601
Stage I	37 (51.4)	177 (47.6)	
Stage II	13 (18.1)	118 (31.7)	
Stage III	20 (27.8)	72 (19.4)	
Stage IV	2 (2.8)	5 (1.3)	

*Statistically significant (*p* < 0.05)

*VATS* Video-Assisted Thoracoscopic Surgery

^a^Data are expressed as number (percent) or median (interquartile range)

The general incidence of postoperative complications did not differ between the PREOP-COV and NON-COV groups, and amounted to 30.6 and 29.3%, respectively (*p* = 0.831).

We found that patients with COVID-19 history more often required re-drainage (6.9 vs. 2.2%, *p* = 0.044) and reoperation (5.6 vs. 1.3%, *p* = 0.042).

The indications for re-drainage in the PREOP-COV and NON-COV groups were residual air space (*n* = 2, 40% vs. *n* = 4, 50%), prolonged air leak (*n* = 2, 40% vs. *n* = 2, 25%) and pleural effusion (*n* = 1, 20 vs. 2, 25%). The indications for reoperation in the PREOP-COV and NON-COV groups were bleeding (*n* = 2, 50% vs. *n* = 4, 80%) and prolonged air leak (*n* = 2, 50 vs. *n* = 1, 20%).

In the PREOP-COV group compared to the NON-COV group, median intraoperative estimated blood loss was higher (150, [IQR, 100–250] vs. 150 [IQR, 50–200] mL, *p* = 0.007) and median postoperative chest tube duration was lower (3 [IQR, 2–4] vs. 3 [IQR, 2–5] days, *p* = 0.008, respectively).

The incidence of other complications and postoperative hospital stay duration did not differ between the groups (Table 3).

**Table 3:** Comparison of outcomes of surgery between patients with (PREOP-COV) and without (NON -COV) history preoperative SARS-CoV-2 infection

Variables^a^	PREOP-COV *n* = 72	NON-COV *n* = 372	*p* value
Complications	22 (30.6)	109 (29.3)	0.831
Prolonged air leak	8 (11.1)	44 (11.8)	0.863
Transfusion	8 (11.1)	24 (6.5)	0.162
Re-drainage	5 (6.9)	8 (2.2)	0.044*
Surgery for complications	4 (5.6)	5 (1.3)	0.042*
Residual air space	5 (6.9)	15 (4.0)	0.345
Atrial arrythmia	3 (4.2)	23 (6.2)	0.783
Atelectasis	0	14 (3.8)	0.140
Pneumonia	1 (1.4)	10 (2.7)	0.243
Surgery for complications	4 (5.6)	5 (1.3)	0.042*
Reintubation	1 (1.4)	2 (0.5)	0.413
Bronchopleural fistula	1 (1.4)	2 (0.5)	0.413
Wound infection	1 (1.4)	1 (0.3)	0.298
Kidney failure	1 (1.4)	2 (0.5)	0.413
Chest tube duration, days	3 (IQR, 2–4)	3 (IQR, 2–5)	0.008*
Hospital stay, days	6 (IQR, 4–7)	6 (IQR, 5–8)	0.058
Alive at discharge	70 (97.2)	370 (99.5)	0.125
Alive at 90-days	70 (97.2)	365 (98.1)	0.644

*Statistically significant (*p* < 0.05)

^a^Data are expressed as number (percent) or median (interquartile range)

Outcomes at discharge and 90-days after surgery did not differ between the groups. Among the PREOP-COV and NON-COV groups, 97.2 and 99.5% were alive at discharge (*p* = 0.125), and 97.2 and 98.1% were alive 90-days after surgery (*p* = 0.644), respectively. The reasons of the in-hospital death in two patients in PREOP-COV group were bronchopleural fistula and sepsis, and in two patients in the NON-COV group, pneumonia resulting in sepsis and pulmonary embolism, respectively.

In the group of patients with a history of COVID-19, we did not find any correlation between the occurrence of postoperative complications and the severity of COVID-19, the occurrence of hospitalization due to COVID-19, and the time from the diagnosis of SARS-CoV-2 infection to surgery (Table 4). None of the patients had a history of Intensive Care Unit treatment due to COVID-19. In 19 of 72 patients (26.4%) with a history of SARS-CoV-2 infection, a lung tumor was first diagnosed in chest X-ray or computed tomography studies performed during treatment or follow-up for COVID-19.

**Table 4:** Comparison of characteristics of the course of preoperative COVID-19 between patients with and without complications after pulmonary lobectomy or segmentectomy

Variables^a^	Complications *n* = 22	No complications *n* = 50	*p* value
Severity of COVID-19			0.816
Asymptomatic to mild	13 (59.1)	31 (62.0)	
Moderate to severe	9 (40.9)	19 (38.0)	
Hospital treatment for COVID-19	3 (13.6)	8 (16.0)	0.797
Time from COVID-19 diagnosis to surgery, weeks	19.8 (4.6–52.1)	17.5 (5.6–78.7)	0.629

^a^Data are expressed as number (percent) or median (range)

## Discussion

The most important finding of the study is that patients who had COVID-19 in the period preceding the surgery did not have a significantly increased risk of complications and in-hospital and 90-day mortality after lung resection compared to patients who have never had SARS-CoV-2 infection. To our knowledge, this is the first study to assess the relationship between the history of COVID-19 and the incidence of complications and early outcomes after anatomical resection for NSCLC.

Studies indicate that surgery in patients who recovered from COVID-19 may be associated with an increased risk of complications. The results of the COVIDSurg-Cancer study showed an increased risk of pulmonary complications and in-hospital mortality in patients operated on for any cancer; the risk was increased especially in the early period after SARS-CoV-2 infection and returned to baseline levels approximately seven weeks after recovery [17]. Because the study included patients with different types of cancer, its results cannot be directly applied to patients operated on for NSCLC. The results of our study indicate that the incidence of complications and mortality does not differ in patients with and without a history of COVID-19. Since most patients were operated on after 7 weeks of COVID-19, we cannot comment on the safety of the operation in the immediate period following SARS-CoV-2 infection. However, considering the results of studies indicating that delaying surgery for more than 8 weeks after the diagnosis of lung cancer is associated with a worse prognosis, we assume that NSCLC surgery in patients who recovered from COVID-19, does not demonstrate pulmonary and/or cardiac symptoms of post-COVID-19 complications, and meet routine eligibility criteria for surgery, should not be postponed [18].

Some authors suggested more accurate preoperative risk estimation, including testing for C-reactive protein or interleukins [19, 20]. However, the results of our study indicate that the standard patient qualification process is sufficient to achieve a low complication rate and good outcomes. Exercise testing should be considered in patients with comorbidities, who demonstrate prolonged signs or symptoms related to prior COVID-19 or have abnormal pulmonary function tests results, according to the existing guidelines [21, 22].

Apart from the incidence of complications and in-hospital mortality, the outcome at 90 days is also considered a good indicator of the surgical treatment quality and a useful endpoint for studies analyzing the risk factors of surgery. Studies in lung cancer surgery have shown that 90-day mortality is about twice as high as in-hospital mortality [23]. Deaths in the early period after hospital discharge are predominantly associated with comorbidities, mainly coronary heart disease, cerebrovascular disease, chronic kidney disease, and chronic obstructive pulmonary disease [24]. In patients after COVID-19, pulmonary, cardiovascular, and thromboembolic complications may persist for a long time after recovery and these factors could theoretically lead to an increase in 90-day mortality following lung surgery [25]. The results of our study indicate that the history of COVID-19 in the preoperative period does not significantly affect the 90-day outcomes. These results should be treated with some caution since the absolute number of patients who died was relatively small. Larger multicenter studies would be needed to more accurately assess the relationship between COVID-19 history and in-hospital and 90-day mortality of lung cancer surgery.

The last findings of the study were that patients who recovered from COVID-19 more often required reoperation and chest tube reinsertion, usually because of prolonged air leak, residual air space, pleural effusion, or bleeding. The reason for this is unclear to us as we did not find any explanation in the literature. In some patients, during surgery, we observed reactive changes in the lymph nodes and an increased density of tissues around the hilum of the lung, similar to those found in inflammatory diseases. Dissection of tissues may be associated with increased pleural fluid production and possible bleeding requiring surgical intervention [26]. When it comes to re-drainage and reoperation resulting from prolonged air leakage and residual air space, the reason could have been the poorer quality of the lung parenchyma in patients after SARS-CoV-2 infection. Prolonged air leak after lung resection has been linked to poorer pulmonary function tests results [27]. It has also been proven that in post-COVID-19 patients, the reduction in lung function may persist for a longer time [28]. Although the existence of this type of relationship is possible, it is purely hypothetical at this stage and requires further in-depth research.

### Limitations

First of all, a limitation of the study could be the inclusion of patients operated at different time intervals in the control and study groups, which could have resulted in a selection bias. However, it prevented the unintentional inclusion of patients with undetected SARS-CoV-2 infection in the control group. The number of infections was estimated to be a few times greater than the number of diagnosed cases [29]. Selecting a control group based on a negative medical history would most likely lead to the inclusion of patients with undiagnosed COVID-19, which could distort the results of the study. In addition, the pre-, intra-, and postoperative surgical procedures did not change over the period covered by the study. Hence significant selection bias is highly unlikely. Secondly, the size of the study group was too small to analyze whether the severity of COVID-19 and the time from recovery from COVID-19 to lung cancer surgery were related to treatment outcomes. However, at this point, we aimed to assess whether the COVID-19 history itself was related to the results of surgical treatment. Another limitation of the study was the lack of data on the SARS-CoV-2 variant. In the period covered by the study, apart from the initial variant of SARS-CoV-2, variants B.1.1.7 (Alpha), B.1.617.2 (Delta) and B.1.1.529 (Omicron) were successively dominant in Poland, but the details regarding the variant in each patient were not available. Some readers may also consider the lack propensity score matching to construct the control group a methodological error of the study. We decided not to use this method because it could have led to imbalance, especially considering that the study and control groups did not differ in general, surgical, and histopathological characteristics.

## Conclusion

We conclude that preoperative SARS-CoV-2 infection is unrelated to the incidence of complications, and outcomes at discharge from the hospital and 90-days after surgery. Prior COVID-19 should probably not be considered a significant risk factor for lung cancer surgery; however, there is a need for a larger multicenter study to confirm these observations.
